# BioSWR – Semantic Web Services Registry for Bioinformatics

**DOI:** 10.1371/journal.pone.0107889

**Published:** 2014-09-18

**Authors:** Dmitry Repchevsky, Josep Ll. Gelpi

**Affiliations:** 1 Barcelona Supercomputing Center, Life-Sciences Department, National Institute of Bioinformatics, Computational Bioinformatics Node, Barcelona, Spain; 2 Department of Biochemistry and Molecular Biology, University of Barcelona, Barcelona, Spain; Huazhong University of Science and Technology, China

## Abstract

Despite of the variety of available Web services registries specially aimed at Life Sciences, their scope is usually restricted to a limited set of well-defined types of services. While dedicated registries are generally tied to a particular format, general-purpose ones are more adherent to standards and usually rely on Web Service Definition Language (WSDL). Although WSDL is quite flexible to support common Web services types, its lack of semantic expressiveness led to various initiatives to describe Web services via ontology languages. Nevertheless, WSDL 2.0 descriptions gained a standard representation based on Web Ontology Language (OWL). BioSWR is a novel Web services registry that provides standard Resource Description Framework (RDF) based Web services descriptions along with the traditional WSDL based ones. The registry provides Web-based interface for Web services registration, querying and annotation, and is also accessible programmatically via Representational State Transfer (REST) API or using a SPARQL Protocol and RDF Query Language. BioSWR server is located at http://inb.bsc.es/BioSWR/and its code is available at https://sourceforge.net/projects/bioswr/under the LGPL license.

## Introduction

To the extent that the number of Web services available to the Life Science community is continuously growing, there is a need to provide better ways for their description, categorization and discovery. Existing Web services catalogues like EMBRACE [1] or BioCatalogue [2] are usually bound to Web Service Definition Language (WSDL) and limit themselves to annotation of service entities. The need to provide a richer way to describe Web services raised an interest for ontology-based models for Web services description. A large variety of Web services types and protocols either limits the scope of such ontologies to concrete type of services or makes them so abstract that still requires WSDL usage. In the latter case, a link between WSDL components and their semantic descriptions is usually done via Semantic Annotations for WSDL and XML Schema (SAWSDL) annotations [3].

WSDL 2.0 brought a new conceptual model with considerable improvements in Representational State Transfer (REST) Web services description. A possibility to describe RESTful Web services along with Simple Object Access Protocol (SOAP) based ones is a clear step forward especially in life science domain where both approaches are intensively used. Another remarkable improvement of WSDL 2.0 was the introduction of Internationalized Resource Identifiers (IRIs) for its described components. The ability to unambiguously identify every WSDL 2.0 entity as a resource allows using these identifiers within ontologies. WSDL 2.0: RDF Mapping specification provides such ontology to express WSDL 2.0 in Web Ontology Language (OWL). The possibility to describe Web services using standard semantic vocabularies, does not replace the need of ontologies for the life science domain, but rather provides a better integration where Web services may be represented in pure semantic way.

BioSWR registry is a response to the need to introduce a standard semantic view into Web services in addition to the traditional WSDL-based one. OWL/RDF representation of service definitions allows using SPARQL Protocol and RDF Query Language (SPARQL) for Web services discovery and annotation, while WSDL-based representation provides a compatibility with existing Web services development tools.

The choice of WSDL 2.0 as a basement for Web services descriptions was dictated by the need to support a broader range of Web services. The required easy bidirectional transformation between representational models puts further limitations to the choice of the Web services description ontology. Within several frameworks and specifications aimed at semantic Web services description, W3C Web Services Description Language (WSDL) Version 2.0: RDF Mapping specification has been chosen as a standard basement for a modern Semantic Web Registry implementation.

## Functionality

**Figure 1:**
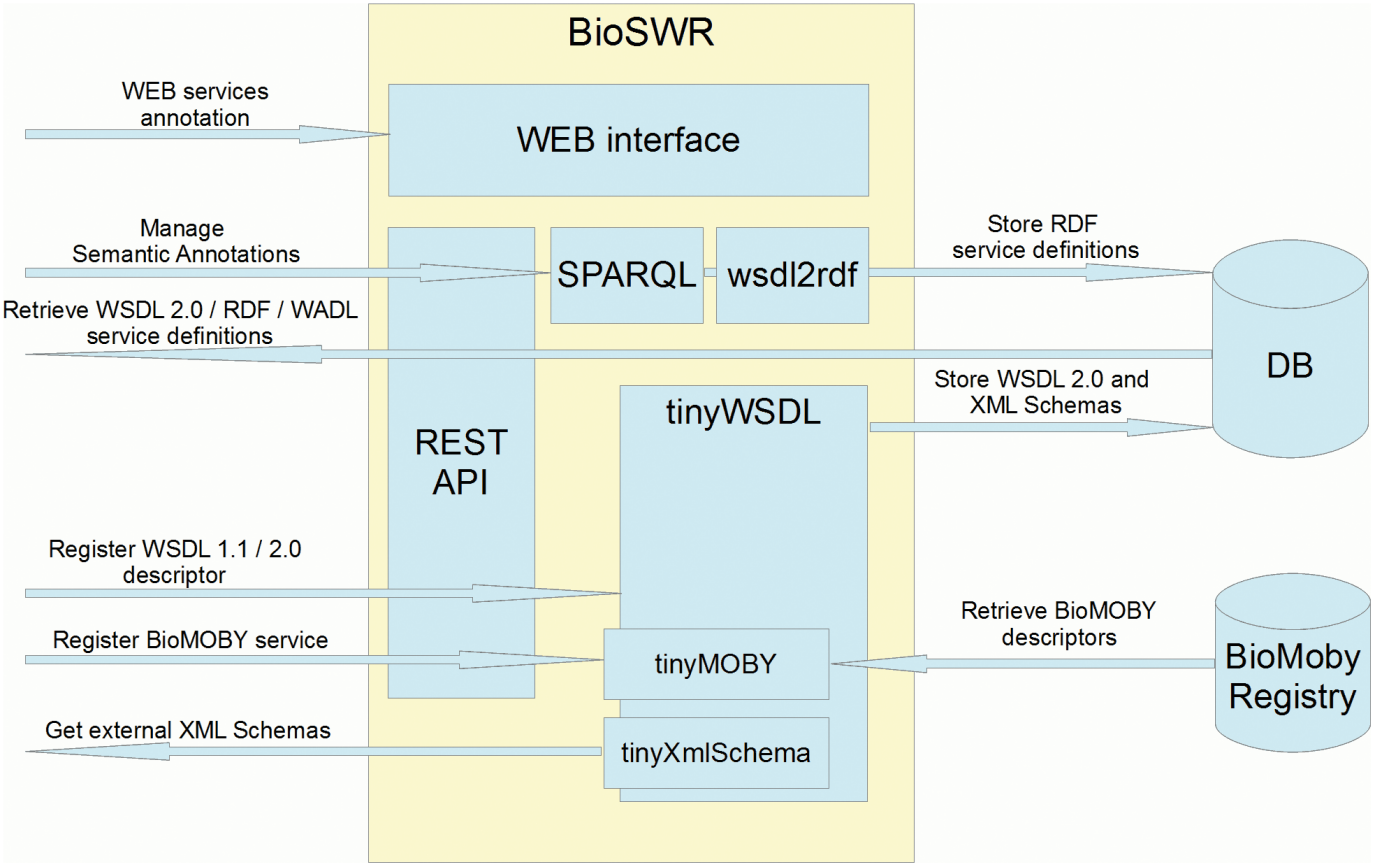
BioSWR general architecture.

BioSWR is designed to support WSDL 1.1/2.0 Web services, providing a local storage for the registered services and dependent XML Schema files (Figure 1). While WSDL is generally used to describe SOAP and sometimes RESTful Web services, WSDL 2.0 component model is protocol agnostic and may be adapted to virtually any type of services. To extend a number of supported Web services, BioSWR provides also support for BioMoby [4] services. In the latter case the support is implemented via SAWSDL extension, embedding ^my^Grid BioMoby semantic model [5] into WSDL 2.0 descriptor. BioSWR also relies on SAWSDL for general services annotation, using EMBRACE Data and Methods (EDAM) [6] ontology as the primary source of semantic annotations.

RESTful services are becoming very popular due to their simplicity of use. For this same reason, unlike SOAP-based web-services, they usually lack formal description and are difficult to integrate into workflows. To help developers in the description, and registration of their RESTful web-services, BioSWR documentation provides a simple WSDL 1.1 template. For those RESTful web-services that may be accessed via web browser, BioSWR also provides a sample URL-based template. Finally, for clients requiring more formal descriptions, a WADL description is provided along with the stored WSDL.

### BioSWR REST API

BioSWR provides a REST-based API to manage Web services storage (Table 1). The API offers HTTP access to stored Web services definitions that can be directly imported into tools like Taverna [7]. While new Web service registration may be performed by any authenticated user, service removal may be accomplished only by a service owner – the user who originally registered the service. Web service owner may also allow other users to annotate the service, keeping the rights to remove inappropriate annotations. Credentials should be provided via standard basic HTTP Authentication [8].

**Table 1:** BioSWR REST Web services API.

HTTP location	HTTP method	Description
/service/register?url = {url}&lsid = {lsid}	GET	Registers the service providing its WSDL file URL or a BioMoby LSID identifier. Returns a WSDL service description.
/service	GET	Get an OWL/RDF ontology with all registered services.
/service/{id}	GET	Get a Web service description by its identifier.
/service/{id}	DELETE	Removes a Web service with given identifier.

### Semantic data querying

**Figure 2:**
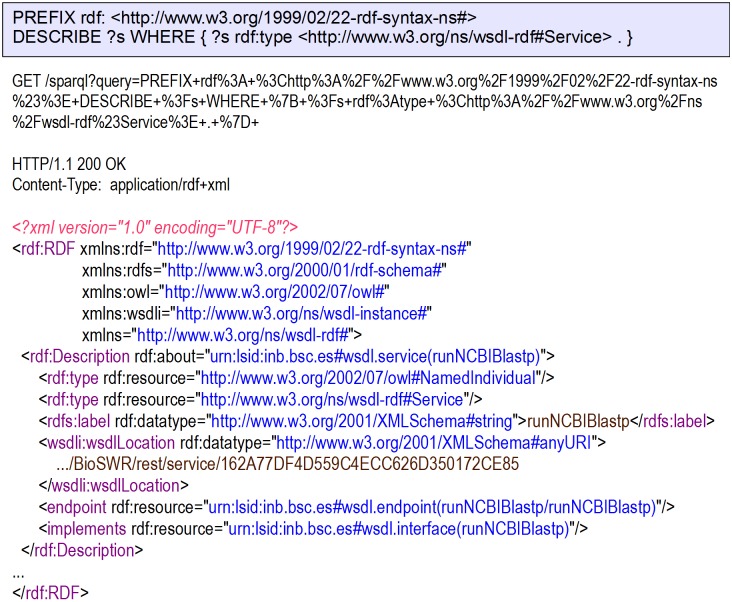
Find all registered Web services via SPARQL DESCRIBE query.

Instead of providing a custom API for Web services search, BioSWR provides SPARQL querying over stored Web services descriptions. BioSWR supports SPARQL 1.1 Protocol query variations via HTTP GET or HTTP POST bindings (Figure 2).

To facilitate SPARQL-based repository discovery, all results are provided with a wsdli:wsdlLocation property to locate the original description document as it was found in the registry. SPARQL query may also be used to filter Web services in the Web interface, however to provide a friendly interface for non-expert users, simple text-based search is available.

**Figure 3:**
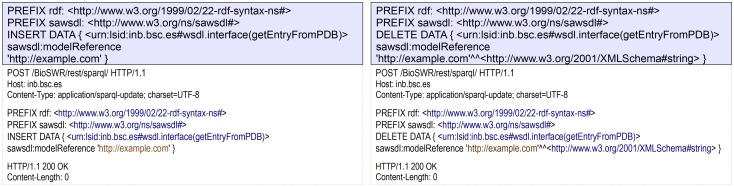
Add/Remove SAWSDL reference via SPARQL UPDATE query.

SPARQL UPDATE support provides a simple programmatic way to manage semantic annotations such as rdfs:comment and SAWSDL references (Figure 3).

### Semantic enrichment

**Figure 4:**
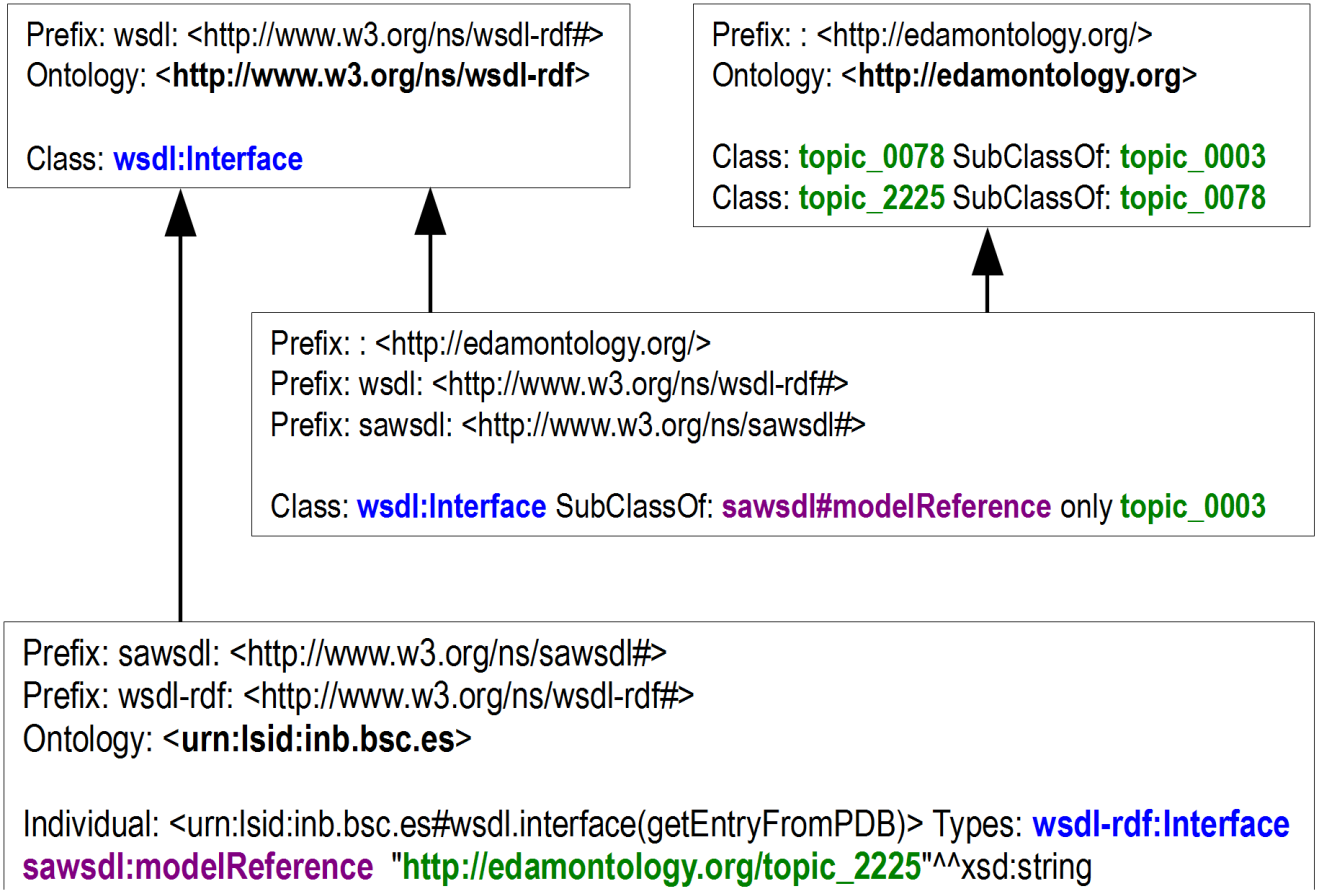
Example of Semantic Rules definitions. Here wsdl: Interface sawsdl#modelReference property is restricted to topic_0003 (Topic). Because topic_2225 (Protein databases) is a subclass of topic_0003, urn:lsid:inb.bsc.es#wsdl.interface(getEntryfromPDB) interface (which is an individual of wsdl: Interface) may be annotated with it without making the ontology inconsistent.

In accordance with SAWSDL specification, semantic enrichment is attained via sawsdl:modelReference attributes. The choice of an appropriate annotation subject is defined internally as logical axioms and realized through semantic reasoning (Figure 4). This approach provides flexibility when choosing external annotation sources. BioSWR provides EDAM ontology integration. EDAM was specially designed for bioinformatics/computational biology domain and provides a wide coverage of common bioinformatics objects and methods.

Apart from SAWSDL references, basic OWL 2 annotation properties such as rdfs:comment, rdfs:seeAlso and rdfs:isDefinedBy are supported. BioSWR keeps track of all annotations, annotating them with rdfs:isDefinedBy (annotation of another annotation). The latter provides flexibility in annotation management, where only authorized authors may modify outdated annotations.

### BioMoby integration

BioMoby has been a widely used framework to deploy general and specialized bioinformatics WS. Although BioMoby usage has declined over the last years, a large number of services still exist. BioSWR provides BioMoby integration through semantically enriched WSDL 2.0 descriptions. While BioMoby services are SOAP-based they use a special BioMoby message format which cannot be expressed in XML Schema. From SOAP point of view its content constitutes an encoded string. Fortunately, BioMoby already provides its own service’s descriptions through BioMoby ^my^Grid ontology. These definitions are integrated into generated BioMoby WSDL 2.0 descriptors and linked with input/output elements via SAWSDL annotations. The tinyMOBY [9] extension of tinyWSDL [10] parser provides ^my^Grid semantic annotations management within WSDL 2.0 descriptions. tinyMOBY allows to extract BioMoby data-type definitions from the embedded ^my^Grid ontology providing a direct integration with BioMoby Java API [11]. The latter eliminates the need of using BioMoby Registries, mostly inactive nowadays, since tinyMOBY datatype definitions includes all required information for BioMoby message preparation and further Web service execution.

### WADL support

For WSDL 2.0 services that are described through HTTP Binding Extension, Web Application Description Language (WADL) descriptors may be also obtained via the BioSWR REST API providing an HTTP “Accept: application/vnd.sun.wadl+xml” header. The WADL descriptor may be also found in the service description panel of the Web interface.

### Web services monitoring

One of the most challenging issues in providing any kind of tool registry in Bioinformatics is to keep track of their availability. There are several levels of Web services monitoring that are usually performed to verify Web services operability. BioSWR implements an availability check inspecting the original Web service description that has been used for the registration. The check is performed periodically or upon user request. The absence of Web service description is interpreted as service withdrawal. Modifications of the original Web service descriptions are detected using cyclic redundancy check (CRC) algorithm [12]. An indication of the status of the service (active, modified, or unavailable) is included in the web interface, and services list can be filtered by such parameter.

## Implementation

BioSWR is implemented using Java EE 6 Platform. Web interface is based on Java Server Faces 2.0 and RichFaces component framework. BioSWR REST API is implemented using Java API for RESTful Web Services. SPARQL protocol implementation is based on openRDF Sesame framework [13]. Registered services are stored in a MySQL database.

Technologies chosen as a ground for the registry became a challenging task of implementation of the latest standards in Semantic Web services. To achieve BioSWR goals several brand-new Java libraries have been developed and contributed to the community:

### wsdl2rdf service description library

Semantic representation of Web services requires a solid tool to provide a mapping between WSDL 2.0 and OWL model representation. The wsdl2rdf library provides an easy and straightforward API for WSDL 2.0 ontology management, hiding OWL complexity from developers. As WSDL 2.0 ontology does not impose most of the restrictions defined in WSDL 2.0 specification, the advantage of the API usage over a straight ontology manipulation is to provide ontology consistency validation. The wsdl2rdf library strictly follows the original ontology provided by the WSDL 2.0 RDF Mapping specification [14] and is based on The OWL API [15].

### WSDL 2.0 parsing library

To parse and manipulate WSDL 2.0 descriptions, a brand new WSDL 2.0 library has been developed. The library is based on WSDL 2.0 Part 1: Core Language [16] and WSDL 2.0 Part 2: Adjuncts specifications [17], and supports both SOAP and HTTP binding extensions. A complementary **tinyXmlSchema** extension library has been developed to provide an easy manipulation of referenced XML Schema elements. The tinyXmlSchema library is based on Apache XML Schema 2.0 library [18]. In addition to standard WSDL 2.0 extensions, tinyWSDL supports SAWSDL annotations.

### WSDL 2.0 BioMoby extension library

In order to provide better integration with BioMoby services, **tinyMOBY** extension library has been developed. The library allows representing BioMoby services through semantically enriched WSDL 2.0 descriptors. Generated descriptors embed ^my^Grid BioMoby RDF definitions that can be used to reconstruct BioMoby message format. As well as the owl2rdf library, tinyMOBY is based on The OWL API. The integration with BioMoby is implemented via lightweight BioMoby Java API.

## Discussion

Extending the EMBRACE and BioCatalogue registries philosophies, BioSWR offers many unique features for Semantic Web Services providers and consumers. The most significant advancement of BioSWR is the adoption of WSDL 2.0 and its standard OWL-based representation. Semantic representation of Web services allows using SPARQL query language for Web services discovery and annotations and greatly simplifies BioSWR REST API eliminating a need in respective methods.

BioMoby was a fairly extended protocol for SOAP-based web services, and a significant number of services are still available. BioSWR offers semantically enriched WSDL 2.0 descriptors for BioMoby services, freeing BioMoby clients from the need to interact with a BioMoby Central, and simplifying BioMoby services execution.

BioSWR WSDL 2.0 support also contributes to bringing RESTful Web services back to the standards. Being very popular to access data from repositories due to its simplicity of use, RESTful Web services are often developed outside of the established standards. This precludes those services from being integrated in bioinformatics, and even makes difficult its usage with current clients without a manual adaptation. BioSWR has been developed with a special interest in such integration. For RESTful Web services BioSWR provides both WSDL and WADL descriptions, which can be used by appropriate clients. BioSWR also facilitates URL-based templates for RESTful retrieve operations (HTTP GET verb). As a proof of concept the complete set of RESTFul services generated from RCSB have been registered in BioSWR, and an example tutorial using this kind of services in combination with classical SOAP-based is provided.

BioSWR pushes bioinformatics Web Services Registries to a new level of semantic support providing Semantic Web Services descriptions based on their standard OWL/RDF representation. In anticipation of greater SWS adoption by life science community, BioSWR facilitates a smooth transition from conventional WSDL 1.1 service definitions to OWL-based WSDL ontology. The use of SAWSDL for semantic annotations provides interoperability with tools that rely on standard WSDL definitions. The support of SPARQL query language for service discovery, a Web 2.0 single page design, along with a traditional REST-based interface, to register, filter and annotate Web services, makes BioSWR a powerful registry for bioinformatics Web services.
